#  Systematic Review of Drug-Related Hospital Admissions: Common Errors in Reporting

**DOI:** 10.30476/BEAT.2020.84167.1059

**Published:** 2020-07

**Authors:** Mohammad Bagher Shamsi, Siavash Vaziri, Hamid Reza Mozaffari, Maryam Mirzaei

**Affiliations:** 1 School of Allied Medical Sciences, Kermanshah University of Medical Sciences, Kermanshah, Iran; 2 Department of Infectious Diseases, School of Medicine, Kermanshah University of Medical Sciences, Kermanshah, Iran; 3 Department of Oral and Maxillofacial Medicine, School of Dentistry, Kermanshah University of Medical Sciences, Kermanshah, Iran

**Dear Editor,**

 We have read with great interest the review entitled “Drug related hospital admissions; A systematic review of the recent literature” by Ayalew and colleagues in Bull Emerg Trauma in 2019, 7th volume and 4th issue [1]. In fact, it seems that this review article has not paid proper attention to the reporting of the paper. We noticed that there were some reporting flaws, which we would like to discuss to improve reporting of this review study.

The first matter is related to the searched databases. Google Scholar is not a database; it is just a search engine. It is good to recognize the difference between these two. It will be better, if the authors specify which one is a database, which one is a search engine, directory, etc.

Google also mentions in the electronic resources that it is a search engine and does not fit into a scientific article, and it is better not to be included in this section. Another point is about the number of searched issues in the international databases. The authors have stated that the aim was to summarize prevalence of hospital admissions due to drug-related problems and the possible prevention of drug related hospital admissions with systematic review worldwide. In searched databases, they addressed only one international database, which can have yielded less publication. 

For prevention to lose a lot of relevant studies in a systematic review, it is highly recommended that all relevant existing papers in one area to be identified. Second, for transparency and reproducibility, the authors should consider addition of search strategies for at least one database, including any limits that could be repeated. In this review, search strategy was not well established [2-4]. Finally, the data regarding the quality assessment of included studies and used checklist was not reported in the methods and the results sections. All should be discussed and be presented both in the methods and the results sections. 

This part lacks the risk of bias within studies. Various tools such as “the Cochrane Collaboration’s tool” for assessing risk of bias in randomized trials and “Joanna Briggs Institute critical appraisal checklist” have been introduced for quality appraisal of quantitative studies that can help the authors in forming objective assessment of the study. If the authors decided to list the quality appraisal scores, ideally it should be presented via text or tables, because the strength of the conclusions drawn in the systematic reviews and metaanalysis are dependent on inclusion of studies that met a minimum standard of quality [5]. Due to substantial increase in the quantity of systematic reviews and meta-analyses in recent years, it is important that reviewers to be familiar with the reporting guidelines such as PRISMA checklist to improve the quality of review reports [3]. 

## References

[1] Ayalew MB, Tegegn HG, Abdela OA (2019). Drug Related Hospital Admissions; A Systematic Review of the Recent Literatures. Bull Emerg Trauma.

[2] Moher D, Liberati A, Tetzlaff J, Altman DG (2009). Preferred reporting items for systematic reviews and meta-analyses: the PRISMA statement. Ann Intern Med.

[3] Moher D, Liberati A, Tetzlaff J, Altman DG (2009). Preferred reporting items for systematic reviews and meta-analyses: the PRISMA statement. J Clin Epidemiol.

[4] Shamsi MB, Arab-Zozani M, Mirzaei M (2019). Methodological Issue on Reporting of Systematic Review of Diagnostic Accuracy of Rapid Ultrasound in Shock. Bull Emerg Trauma.

[5] Bashir Y, Conlon KC (2018). Step by step guide to do a systematic review and meta-analysis for medical professionals. Ir J Med Sci.

